# New frontiers in the p-hacking practices: assessing the effect of data peeking practice on type-I error rate through computational modeling

**DOI:** 10.3389/fpsyg.2026.1769309

**Published:** 2026-09-08

**Authors:** Francesca Freuli, Stefano Noventa, Luigi Lombardi

**Affiliations:** 1 Department of Psychology and Cognitive Science, University of Trento, Trento, Italy; 2 University Centre of Statistics in the Biomedical Sciences, Vita-Salute San Raffaele University, Milan, Italy; 3 Department of General Psychology, University of Padua, Padua, Italy; 4 IRCCS San Camillo Hospital, Venice, Italy

**Keywords:** data-peeking, monte-carlo simulation, p-hacking, replicability crisis, type-I error rate

## Abstract

Data-peeking, the practice of stopping data collection once significant results are obtained, poses a significant threat to the credibility of research by increasing the likelihood of observing and publishing false positive results. The effect of this practice on scientific reliability has traditionally been assessed through Monte-Carlo simulations, which suffer from some methodological issues (e.g., Monte–Carlo standard error is often overestimated). This paper presents a computational model that, through a series of convolutions between normal and truncated normal distributions, estimates the increase in false-positive rates due to data-peeking practice, overcoming the issues of the simulation method. The performance of the computational model was compared against Monte-Carlo simulations under different conditions, including alpha levels (0.05, 0.025, 0.01, and 0.001), sample sizes (40, 80, 160, and 320), and numbers of interim analyses (1 to 10). The results confirmed the computational model's accuracy and reliability, showing that computational values fell within two standard deviations of the simulated means. In conclusion, the proposed model not only presents an faster and error-reducing approach to estimating the effects of data-peeking practice but also provides guidelines for the development of similar models to examine other forms of p-hacking, thereby improving the understanding and study of their effects.

## 1 Introduction

Data-peeking (DP-Strube, 2006), also known as Questionable Interim Analysis, is a specific form of p-hacking practice that increases the probability of observing a significant result in experimental research. While the standard approach for hypothesis testing concerns a single analysis performed on a single data sample, DP practice involves performing multiple hypothesis tests as new data are collected. Specifically, a new test is carried out each time new observations are collected to increase the sample size. This practice is questionable because the data collection-stopping rule depends on the interim analysis result: when the analysis is significant, the data collection is stopped; otherwise, additional data are collected (Nagy et al., 2025). DP can be framed within the broader framework of p-hacking and Questionable Research Practices (QRPs), which involves practices such as HARKing, cherry-picking, subgroup analyses, and, more generally, practices that exploit flexibility in research methodologies (Wicherts et al., 2016). Although these practices differ in their specific mechanisms, they share the common feature of increasing researchers' degrees of freedom and, consequently, the probability of obtaining statistically significant findings. From a statistical perspective, many of these practices can be interpreted as forms of implicit or explicit multiple testing, as researchers repeatedly evaluate different analyses, datasets, or stopping rules before reporting the final result. The problem of controlling false positive rates under multiple testing has a long history in statistics, particularly within the Family-Wise Error Rate (FWER) framework. Methods such as the Bonferroni correction, Holm's sequential procedure, and Tukey's range test were developed to control the probability of observing at least one false positive result across a family of statistical tests (Holm, 1979; Hochberg and Tamhane, 1987; Shaffer et al., 1995). These procedures are specifically designed to maintain appropriate control of the type-I error rate during repeated or interim analyses and therefore represent standard statistical practice in sequential testing contexts. By contrast, DP practices involve performing repeated interim analyses without applying corrections to the nominal significance threshold, thereby increasing the probability of obtaining false positive results. In this sense, DP can be interpreted as an uncorrected form of sequential testing that may contribute to the inflation of false positive findings in the scientific literature. Given the obvious connection between DP practices and the replicability crisis, some studies applied Monte Carlo simulation methods to assess how DP affects the growth of false positive results in the literature (Simmons et al., 2011; Roettger, 2019; Wang et al., 2017). Although simulation approaches provide a flexible framework for studying complex scenarios, they may still present important methodological challenges related to Monte Carlo standard error, computational efficiency, and the stability of simulation-based estimates (Browne et al., 2025). The lack of a unified and standardized methodology for these studies might lead to dangerous biases, thus increasing the time required to run these types of simulations. Therefore, an alternative research method to simulation is needed due to the methodological challenges of simulation studies and to the relevance of understanding the effect of DP on data analysis results.

Simulation studies have been extensively used to evaluate the growth of the type-I error rate due to DP practices. These studies have examined both the number of interim analyses and the increase in sample size added between the two analyses (Simmons et al., 2011; Simonsohn et al., 2014; Armitage et al., 1969). Additionally, since more than one p-hacking practice can be implemented at the same time, these simulated studies have investigated the combined effect of multiple practices on experimental results (Bakker et al., 2012). They have shown that the type-I error rate grows more when more interim analyses with smaller increases in sample size are considered (Stefan and Schönbrodt, 2023). This aspect is particularly relevant in light of the broader literature on the replicability crisis, which has highlighted how underpowered studies are more vulnerable to unstable statistical estimates, inflated effect sizes, and false positive findings (Ioannidis, 2005, 2008; Button et al., 2013). In this context, repeated interim testing performed on small samples may further amplify random fluctuations in the data, thereby increasing the probability of obtaining statistically significant but poorly replicable results. More generally, it is worth noting that a researcher has the certainty of obtaining a significant result by collecting an infinite number of observations and performing an interim analysis after that each new data point is added to the sample (Armitage et al., 1969). This aspect is particularly problematic in the context of isolated single-study findings, where the absence of independent replications may make it difficult to distinguish true effects from results generated through repeated interim testing. Recent methodological literature has highlighted the limitations of interpreting isolated single-study findings in the absence of cumulative evidence and replication, particularly because such findings may encourage overreliance on statistical significance rather than the evaluation of evidence across multiple studies (McShane and Böckenholt, 2017). By contrast, findings based on multiple studies, replications, or meta-analytic evidence may partially mitigate the influence of isolated false positive findings by allowing researchers to evaluate the consistency and directional convergence of effects across independent datasets (McShane and Böckenholt, 2017; Wegener et al., 2024; Head et al., 2015). Indeed, cumulative evidence frameworks make the distribution and variability of observed effects across studies more transparent. Under a true null effect, statistically significant findings generated primarily through DP practices would be expected to show greater directional heterogeneity across studies than would be expected under a genuine non-zero effect (Fabrigar and Wegener, 2016). Nevertheless, DP practices may still influence the broader scientific literature through the accumulation of biased or selectively reported individual studies, potentially affecting the interpretation of cumulative evidence (Ioannidis, 2008). Because DP practices increase the probability of obtaining statistically significant findings, they may also indirectly increase the probability that these results are published due to publication bias phenomenon and subsequently incorporated into meta-analytic evidence synthesis. Consequently, when cumulative evidence frameworks disproportionately include findings affected by DP practices, meta-analytic estimates may progressively reflect distorted patterns of evidence rather than stable underlying effects, particularly when the underlying distribution of published effects no longer adequately reflects the true distribution of empirical findings (Head et al., 2015). Given the impact of DP practices on the reliability of the scientific literature, it is crucial to pay a closer attention to the methodology of simulation studies that, because of non-convergence or other computational issues, don't always yield reliable results (Pawel et al., 2025). Simulations studies have indeed two main drawbacks: (a) the methodological complexity involved in incorporating multiple factors in complex scenarios (Browne et al., 2025), and (b) the challenge of defining the required simulation size (Morris et al., 2019). Because the computational resources and the running-time required by simulations increase substantially in complex scenarios, the number of relevant parameters (e.g. number of interim analyses) that can be explored is often limited, and a comprehensive evaluation of the effect of these parameters is difficult. DP simulation studies usually consider the following simulation design factors: the number of interim analyses (typically from 1 to 10), the total sample size, and the increase in sample size between two interim analyses (usually given by the ratio between the total sample size and the total number of interim analyses). However, to reduce computation time, researchers may underestimate the number of simulations needed, leading to unreliable results (Pawel et al., 2024). Furthermore, a rigorous experimental design requires a priori scheduling of the parameters involved in an experimental protocol (Morris et al., 2019). In this sense, it is important to establish a clear protocol to prevent both intentional and unintentional use of QRPs in simulation studies (Siepe et al., 2024). Indeed, like the QRPs in traditional experimental studies, the QRPs in simulation protocols involve different phases of the study that can bias the results and conclusions (Nieážl et al., 2022). Some of the QRPs are similar to those already acknowledged in the context of experimental designs, such as a lack of clear definition of study objectives or the selective reporting of results that support the hypotheses. However, other QRPs are specific to the simulation context, such as a lack of justification for the number of simulations or the choice of the data generation process. All these practices undermine the replicability of studies because they are implemented to obtain a specific result but often remain unreported. To address all these issues, we propose a novel model for calculating the growth of the type-I error rate resulting from DP practice. Instead of being based on simulations, the model calculates the actual error rate by using the equations describing the DP practice. To build the model we first identify the equation representing the outcome generated through DP, and then reformulate this expression in terms of probability density functions. The resulting computational model solves the identified equation going beyond the limitations of simulation methodology, providing reliable results without the need to define the number of simulations. In this context, the term computational model refers to a numerical/computational solution to the equations capturing the DP practice. Following the literature (Simmons et al., 2011; Stefan and Schönbrodt, 2023) our model estimates the growth of the type-I error rate by applying one-sample t-test and assuming that all data are sampled from a standardized normal distribution.

The present manuscript is structured as follows: in Section 2, we describe a model based on a computational approach and we validate the model through a simulation study. Then, in Section 3, we present the main results about the model's validation. Finally, in Section 4, we discuss the results, highlight the research limitations, and provide suggestions for future work.

## 2 Method

In this section, we first introduce the formal aspects of the DP practice which is essential for understanding the computational model. Subsequently, we discuss the development and validation of the model.

### 2.1 Notation

In this subsection, we present a concise overview of the one-sample *t*-test and formally introduce DP strategies, providing the basis needed to understand the proposed model.

The one-sample *t*-test is the simplest test in the *t*-test family. It is used to test if the population mean μ of a sample drawn from a normally distributed population is, for example, equal to a specific value μ_0_ (that is to say: *H*_0_:μ = μ_0_). The one-sample *t*-test is commonly used to investigate the effect of p-hacking practices in simulation studies (Stefan and Schönbrodt, 2023; Freuli et al., 2023). Let **x** be a random sample of *n* independent and identically distributed (i.i.d.) observations drawn from a normal distribution with unknown mean μ and variance σ^2^. It is not difficult to show that the one-sample *t*-test reduces to


$$\begin{array}{l} t=\frac{\bar{z}}{1/\sqrt{n}} \end{array}$$ (1)

where


$$\begin{array}{l} \bar{z}=\frac{\left(\bar{x}-\mu_{0}\right)}{s} \end{array}$$ (2)

is the standardized sample mean of **x** and *s* denotes its sample standard deviation, as shown in Equation 2.

In DP practice, data are repeatedly analyzed during the data collection, which is stopped as soon as a significant result is observed. For the purposes of this computational model, all observations are assumed to be standardized as defined above. Furthermore, we denote by **z**_*h*_ (1 ≤ *h* ≤ *k*) the standardized samples collected sequentially, while the combined sample used for the *h*-th interim analysis is denoted by **y**_*h*_ and is defined recursively as


$$\begin{array}{l} {\text{y}}_{1}={\text{z}}_{1}\;\text{and}\;{\text{y}}_{h}=\left({\text{y}}_{h-1},{\text{z}}_{h}\right),\;\text{for}h\geq 2. \end{array}$$ (3)

For instance, the researcher starts collecting a sample **y**_1_ = **z**_1_ of *n*_1_ standardized observations. If the obtained result is significant *p*_1_ < α the researcher tries to publish the result otherwise when *p*_1_≥α a new set of standardized observations **z**_2_ is added to the previous one to obtain a new combined sample **y**_2_ = (**y**_1_, **z**_2_), with overall sample size *n*_2_. The data collection stops if the one-sample t-test yields a statistically significant result *p*_2_ ≤ α. In this example, the DP practice consists in two separate analyses: the first is an interim analysis computed on the first sample of *n*_1_ observations while the second is the final analysis computed on the combined sample with size *n*_2_.

Generally, let *k* be the total number of analyses (with *k*−1 interim analyses) performed on the total sample *y*_*k*_ of *n*_*k*_ independent observations, we define a DP strategy, *S*_*k*_ as:


$$\begin{array}{c} S_{k}=\langle \left(n_{1},n_{2},\ldots ,n_{h},\ldots ,n_{k}\right),k\rangle ,\\ \text{s.t.}\;n_{1}<n_{2}<\ldots <n_{h}<\ldots <n_{k} \end{array}$$ (4)

where the interim analysis *h* and the final analysis *k* are performed on sample sizes *n*_*h*_ (with 1 ≤ *h*<*k*) and *n*_*k*_, respectively. So, for instance (following Equation 4), the strategy *S*_2_ = 〈(*n*_1_, *n*_2_), 2〉, involves two analysis performed on sample sizes *n*_1_ and *n*_2_ such that *n*_1_<*n*_2_.

Furthermore, let *n*_*h*−1_ and *m*_*h*_ = *n*_*h*_−*n*_*h*−1_ be the sample sizes of **y**_*h*−1_ and **z**_*h*_, respectively. By assuming that a one-sample t-test is applied, each analysis result ${\bar{y}}_{h}$ (1 ≤ *h* ≤ *k*) is obtained by the weighted average of its sub-samples ${\bar{y}}_{h-1}$ and ${\bar{z}}_{h}$ as in the following equation


$$\begin{array}{l} {\bar{y}}_{h}=\frac{n_{h-1}}{n_{h}}{\bar{y}}_{h-1}+\frac{m_{h}}{n_{h}}{\bar{z}}_{h},\;h=2,\ldots ,k. \end{array}$$ (5)

Moreover the mean


$$\begin{array}{l} {\bar{y}}_{h}=t_{h}\cdot \frac{1}{\sqrt{n_{h}}} \end{array}$$ (6)

where *t*_*h*_ represents the *t*-value obtained by conducting a one-sample *t*-test on the standardized sample **y**_*h*_ (as derived from Equation 1).

Finally, defining the boundaries


$$\begin{array}{l} d_{h}^{+/-}=t_{h}^{+/-}\cdot \sqrt{\frac{1}{n_{h}}}\;\left(1\leq h\leq k\right) \end{array}$$ (7)

and $t_{h}^{+/-}$ as the critical *t*-value with *n*_*h*−1_ degree of freedom (assuming the one-sample *t*-test application), two main events define each *S*_*k*_ strategy:

a) all the results of each intermediate interim analysis *S*_*h*_, with 1 ≤ *h*<*k*, are non-statistically significant, formally

$$\begin{array}{lll} {\bar{y}}_{h} & \in & D_{h}=\left[d_{h}^{-},d_{h}^{+}\right],\;h=1,\ldots ,k-1 \end{array}$$ (8)

where *D*_*h*_ is the domain of non-statistically significant outcomes with boundaries $d_{h}^{-}$ and $d_{h}^{+}$ (as defined in Equations 6 and 7);

(b) the final analysis *k* provides a result ${\bar{y}}_{k}$ included into a domain

$$D_{k}=]-\infty ,d_{k}^{-}[\cup ]d_{k}^{+},+\infty [$$ (9)

of significant outcomes.

### 2.2 Estimating type-I error rate with computational models

In this subsection, we present the computational model to estimate the potential increase in type-I error (TIE) rates resulting from DP practices. We describe the procedure for an increasing number of interim analyses, starting with a simple toy example with one interim analysis and then generalizing the model to incorporate *k*−1 interim analyses (see the DP strategy definition in Equation 4). The proposed model assumes the null hypothesis of no effect and the application of a one-sample *t*-test. These assumptions are shared with the studies reported in the literature (Simmons et al., 2011; Roettger, 2019; Wang et al., 2017; Stefan and Schönbrodt, 2023).

The computational model is composed of two main steps: in the first step it estimates the probability density function (pdf) $f\left({\bar{y}}_{h}\right)$ of the results ${\bar{y}}_{h}$ obtained at each interim analysis *h*; in the second step it uses this pdf to estimate the TIE value. The pdf $f\left({\bar{y}}_{h}\right)$ required in the first step is estimated considering the DP practice definition reported in subsection 2.1. When considering the toy example of a single interim analysis (*k* = 2), in order to estimate the distribution of the final result ${\bar{y}}_{2}$ we must consider the distributions of its two components:


$$\begin{array}{l} {\bar{y}}_{1}\sim N\left(0,\frac{1}{m_{1}}\right)\;\text{and}\;{\bar{z}}_{2}\sim N\left(0,\frac{1}{m_{2}}\right) \end{array}$$ (10)

Given Equation 5 two new variables ${\bar{y}}_{1}^{*}$ and ${\bar{z}}_{2}^{*}$ are defined such that


$$\begin{array}{l} {\bar{y}}_{1}^{*}={\bar{y}}_{1}\frac{m_{1}}{n_{2}}\;\text{and}\;{\bar{z}}_{2}^{*}={\bar{z}}_{2}\frac{m_{2}}{n_{2}} \end{array}$$ (11)

The distribution for the new variable ${\bar{y}}_{1}^{*}$ is a truncated normal density, whereas the distribution for ${\bar{z}}_{2}^{*}$ is simply a normal density:


$$\begin{array}{l} {\bar{y}}_{1}^{*}\sim \mathrm{NT}\left(0,\sigma_{1}^{*2},d_{1}^{-},d_{1}^{+}\right)\;\text{and}\;{\bar{z}}_{2}^{*}\sim N\left(0,\sigma_{2}^{*2}\right) \end{array}$$ (12)

where


$$\begin{array}{l} \sigma_{1}^{*2}={\left(\frac{m_{1}}{n_{2}}\right)}^{2}\left(\frac{1}{m_{1}}\right),\;\sigma_{2}^{*2}={\left(\frac{m_{2}}{n_{2}}\right)}^{2}\left(\frac{1}{m_{2}}\right), \end{array}$$ (13)

and with $d_{1}^{-}$and $d_{1}^{+}$ being the bounds for ${\bar{y}}_{1}$ (Equation 8). At this first step with one interim analysis, we first compute in R framework (R Core Team, 2021) the pdfs $f\left({\bar{z}}_{2}^{*}\right)$ and $f\left({\bar{y}}_{1}^{*}\right)$ where the latter is truncated by restricting its domain to the interval of non-significant results. These computations are implemented in R (R Core Team, 2021) for the availability of suitable built-in functions, though they can be implemented in any statistical software environment. Then we compute the distribution of the final result ${\bar{y}}_{2}$ by the convolution between the distribution of its components. Since significant results at different stages are mutually exclusive, the growth of the TIE rate is defined by the sum of both the α values obtained at the two analysis levels. So the


$$\begin{array}{l} \text{growth of the TIE rate}=\alpha_{1}+\alpha_{2} \end{array}$$ (14)

where α_1_ is the significant level obtained without interim analysis and α_2_ is defined by the integral of $f\left({\bar{y}}_{2}\right)$ between −∞ and the critical mean point ${\bar{y}}_{c_{2}}$.

Now building on the derivation shown in Equations 10–14, we can generalize the process for more than one interim analyses. Following Equation 5, the mean ${\bar{y}}_{h}$ (3 ≤ *h* ≤ *k*) is defined by the weighted mean of its components ${\bar{y}}_{h-1}$ and ${\bar{z}}_{h}$. By defining


$$\begin{array}{l} {\bar{z}}_{h}^{*}=\frac{m_{h}}{n_{h}}{\bar{z}}_{h}\;\text{and}\;{\bar{y}}_{h-1}^{*}=\frac{n_{h-1}}{n_{h}}{\bar{y}}_{h-1} \end{array}$$ (15)

the probability density function $f\left({\bar{z}}_{h}^{*}\right)$ is a normal distribution with mean 0 and variance $\sigma_{h}^{*2}={\left(\frac{m_{h}}{n_{h}}\right)}^{2}\frac{1}{m_{h}}$ (where $\frac{1}{m_{h}}$ is the mean error standard squared), while $f\left({\bar{y}}_{h-1}^{*}\right)$ is computationally re-scaled as $f({\bar{y}}_{h-1}\cdot \frac{n_{h-1}}{n_{h}})$. The computational model derives the probability density function $f\left({\bar{y}}_{h}\right)$ from the convolution between the distributions $f\left({\bar{z}}_{h}^{*}\right)$ and $f\left({\bar{y}}_{h-1}^{*}\right)$ (where ${\bar{y}}_{h-1}^{*}={\bar{y}}_{h}-{\bar{z}}_{h}^{*}$) where the latter is truncated by restricting its domain to the interval of non-significant results (event a). So the probability density function $f\left({\bar{y}}_{h}\right)=f\left({\bar{y}}_{h-1}^{*}+{\bar{z}}_{h}^{*}\right)$ is given by:


$$\begin{array}{l} f\left({\bar{y}}_{h}\right)=\int_{{\bar{y}}_{h}-d_{h-1}^{+}}^{{\bar{y}}_{h}-d_{h-1}^{-}}f\left({\bar{y}}_{h}-{\bar{z}}_{h}^{*}\right)f\left({\bar{z}}_{h}^{*}\right)d{\bar{z}}_{h}^{*}. \end{array}$$ (16)

This integral can be computationally solved through the approxfun() and integrate() R functions.

Lastly, the probability density function $f\left({\bar{y}}_{h-1}\right)$ is also computed by the convolution between its components functions $f\left({\bar{y}}_{h-2}^{*}\right)$ and $f\left({\bar{z}}_{h-1}^{*}\right)$ (as explained for $f\left({\bar{y}}_{h-1}^{*}\right)$ and $f\left({\bar{z}}_{h}^{*}\right)$). So, each $f\left({\bar{y}}_{h}\right)$ is obtained recursively through *h* convolution processes which are solved computationally.

At each interim analysis 1 ≤ *h* ≤ *k*, the significance level α_*h*_ is defined as the integral of $f\left({\bar{y}}_{h}\right)$ between −∞ and the critical mean point ${\bar{y}}_{c_{h}}$ (mean associated with the critical *t* value):


$$\begin{array}{l} \alpha_{h}=\int_{-\infty }^{{\bar{y}}_{c_{h}}}f\left({\bar{y}}_{h}\right)\;1\leq h\leq k \end{array}$$ (17)

Since significant results at different stages are mutually exclusive, the growth of the TIE rate is simply the sum of all α_*h*_ obtained at all interim analysis levels, that is


$$\begin{array}{l} \text{Growth of the TIE rate}=\overset{k}{\underset{h=1}{\sum }}\alpha_{h}. \end{array}$$ (18)

### 2.3 Estimating type-I error rate with Monte-Carlo simulation study

The performance of the models in estimating the increase of TIE rates, was evaluated with a Monte-Carlo simulation approach. To achieve a Monte-Carlo error lower than .5%, we planned 100,000 simulations for each simulation design cell (Morris et al., 2019). All samples were drawn from a normal distribution with mean 0 and standard deviation 1. In order to assess the null hypothesis, a one-sample t-test was performed. We started by sampling *m* observations and applied the statistical test on the resulting sample **y**_1_ = (*y*_1_, …, *y*_*m*_). If the result ${\bar{y}}_{1}$ was statistically significant the simulations stopped and the ${\bar{y}}_{1}$ value was stored. Otherwise, *m* new observations were sampled and added to the previous sample to obtain a new sample **y**_2_ with 2*m* observations. Next, if the analysis performed on **y**_2_ were not significant, *m* new observations were sampled; otherwise, the ${\bar{y}}_{2}$ value was retained and the new simulation started. Each simulation stopped as soon as a significant result was observed (following the definition in Equation 9) or the total number of interim analyses *k* was reached. At the end of the simulation process, probability density functions $f\left({\bar{y}}_{h}\right)$ (with 1 ≤ *h* ≤ *k*) were returned. Like in the computational model, the $f\left({\bar{y}}_{h}\right)$ distribution was used to estimate the growth of the TIE rate at step *h* (Equations 17 and 18).

### 2.4 Simulation study design

In line with what reported in the literature (see, e.g., Simmons et al., 2011; Roettger, 2019), we considered the following three factors in the simulation study design: a) total sample size *n* with four different levels: 40, 80, 160, 320; b) number of analysis *k* with ten different levels: 1, …, 10 (where *k* = 1 means that no interim analysis was performed); c) TIE probability α with four levels: .001, .01, .025, .05. Hence, we obtained a factorial design with a total of 160 = 4 × 10 × 4 design cells. For both models, *m* = *n*/*k* was defined as the number of participants added between two consecutive interim analyses. Since the sum of all *m* has to be equal to *n*, when *m* was not an integer, it was rounded up for the first values and rounded down for the remaining ones.

The simulation studies were carried out in R framework (R Core Team, 2021) using a Google virtual machine VM n2-highcpu-80 with 80 vCPU and 80 GB RAM, Intel Cascade Lake. The R packages parallel (R Core Team, 2021), foreach (Daniel et al., 2022b) and doParallel (Daniel et al., 2022a) were used to parallelize each experimental cell.

## 3 Results

**Figure 1:**
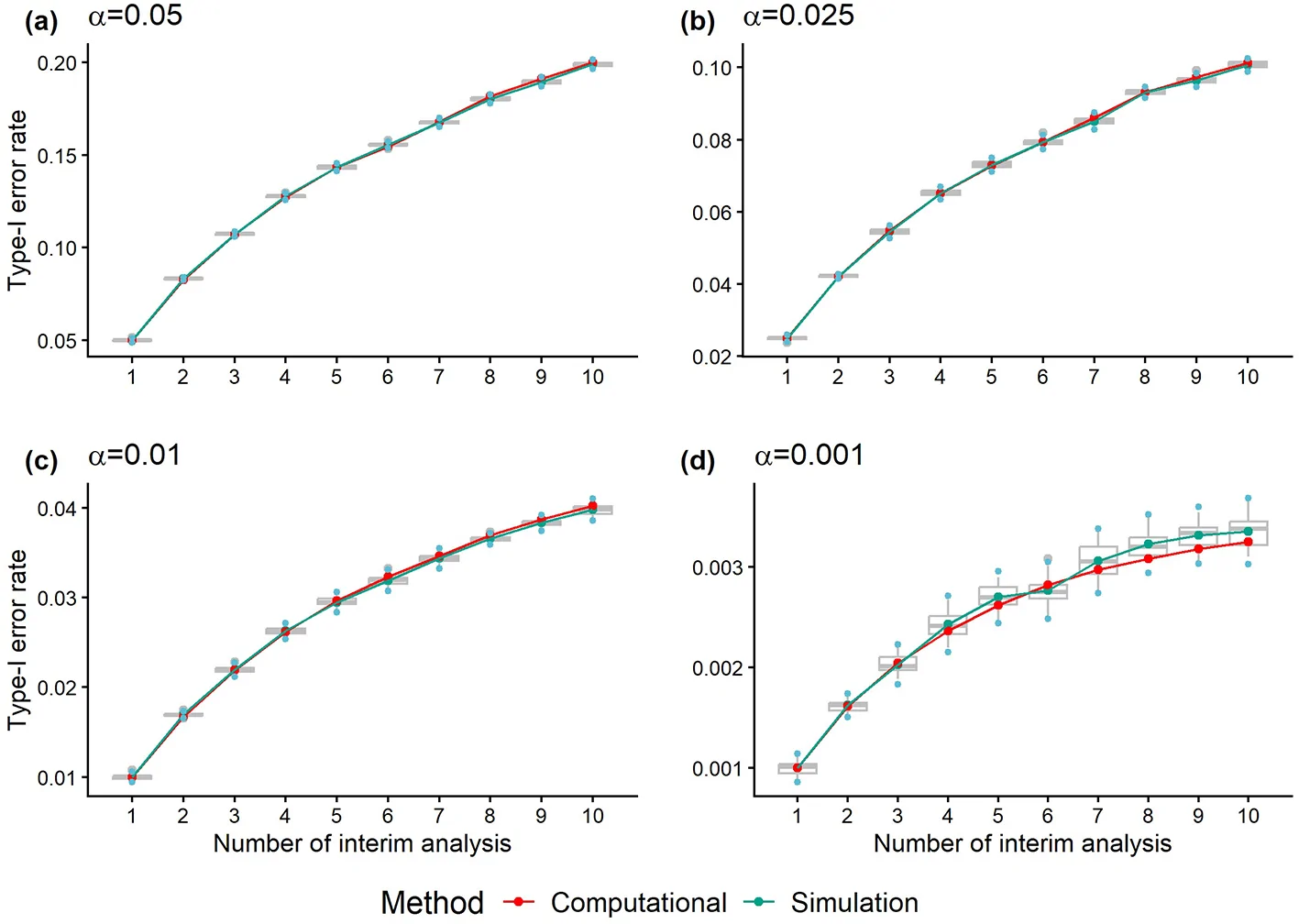
Computational and simulation models' performance for the *n* = 40 condition. The box plots and the green line indicate the simulated points and their mean values. The two blue dots represent the two standard deviations from the simulated mean, while the red lines indicate the computational points. **(a)** α = 0.05; **(b)** α = 0.025; **(c)** α = 0.01; **(d)** α = 0.001.

**Figure 2:**
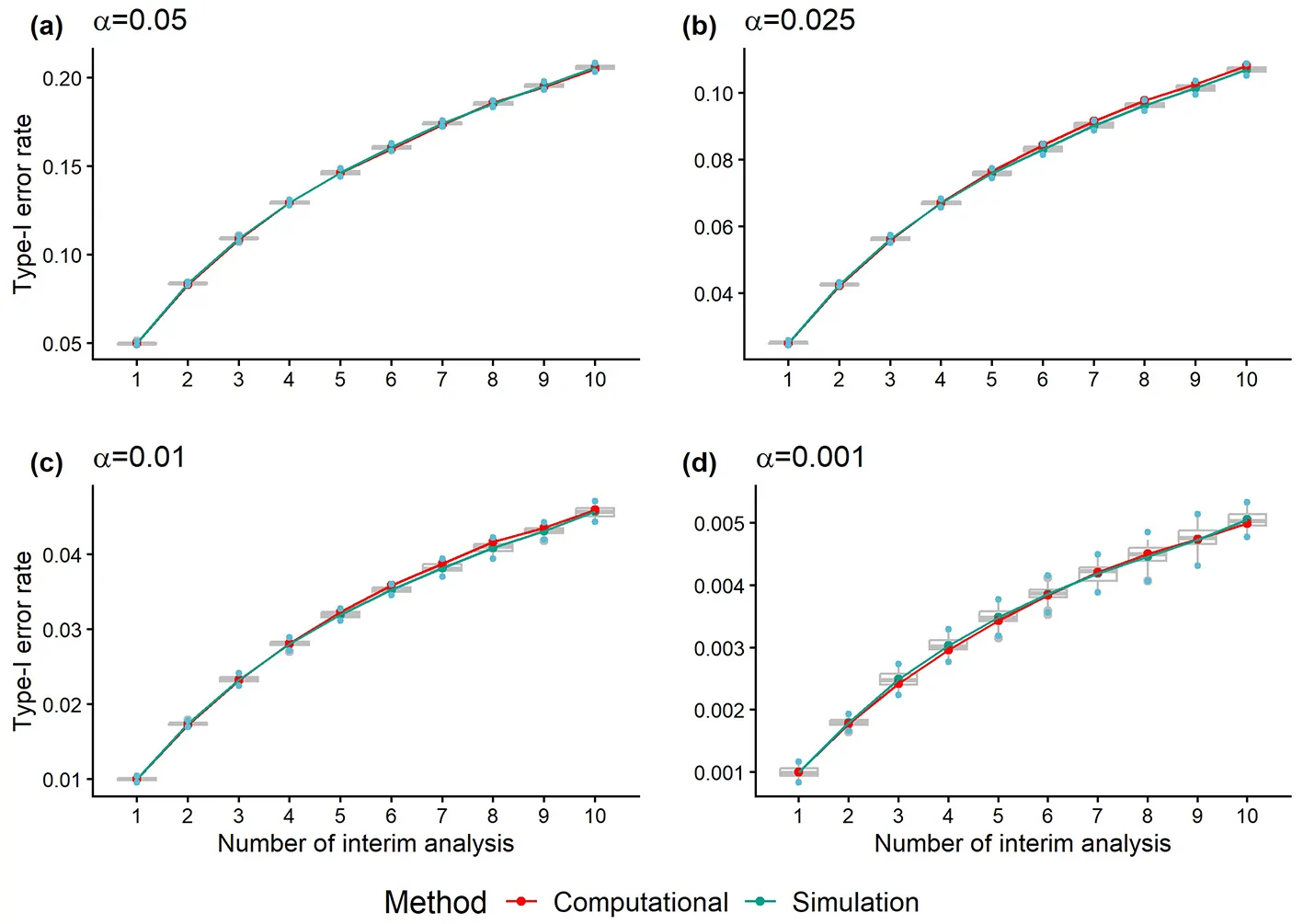
Computational and simulation models' performance for the *n* = 80 condition. The box plots and the green line indicate the simulated points and their mean values. The two blue dots represent the two standard deviations from the simulated mean, while the red lines indicate the computational points. **(a)** α = 0.05; **(b)** α = 0.025; **(c)** α = 0.01; **(d)** α = 0.001.

**Figure 3:**
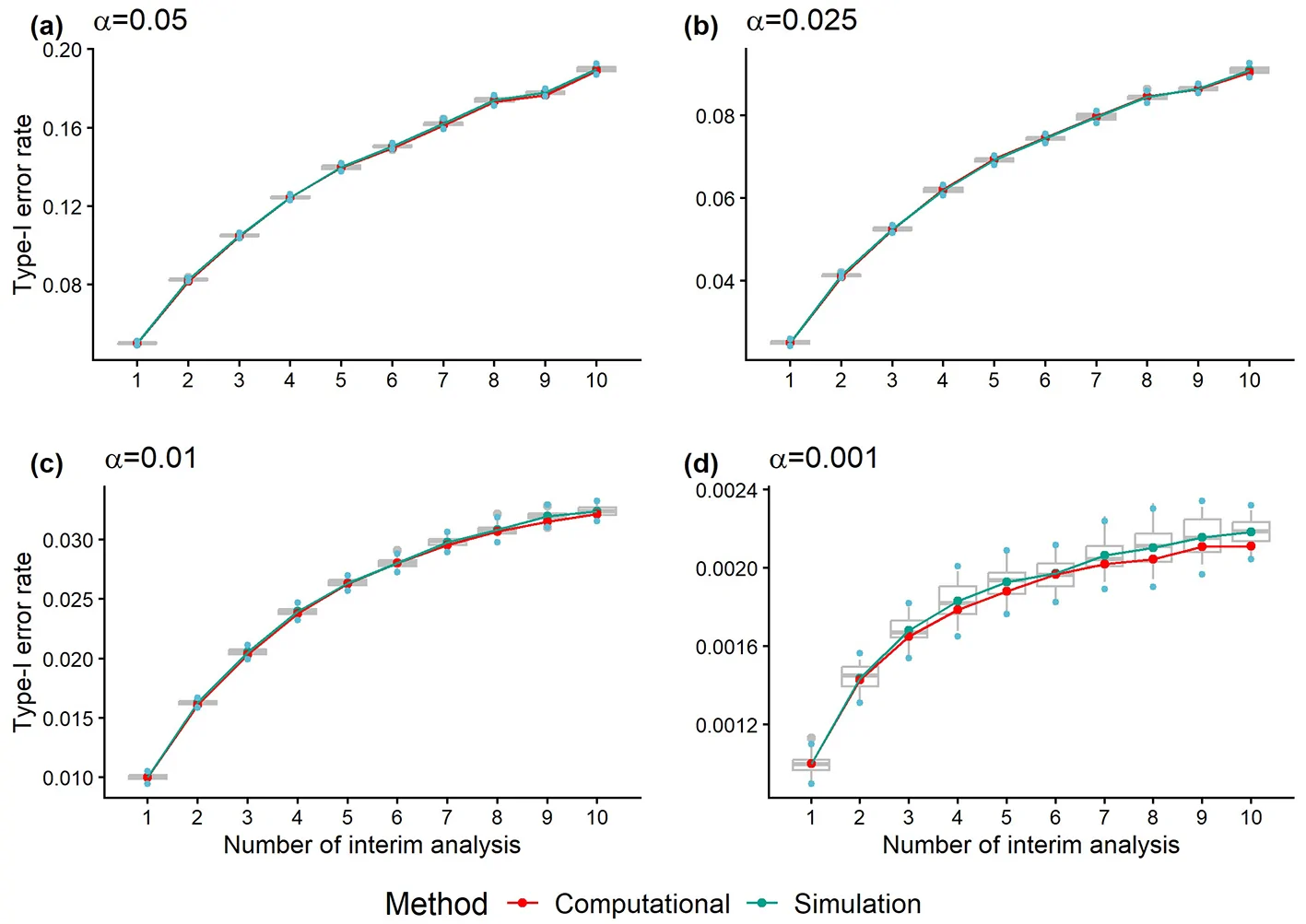
Computational and simulation models' performance for the *n* = 160 condition. The box plots and the green line indicate the simulated points and their mean values. The two blue dots represent the two standard deviations from the simulated mean, while the red lines indicate the computational points. **(a)** α = 0.05; **(b)** α = 0.025; **(c)** α = 0.01; **(d)** α = 0.001.

**Figure 4:**
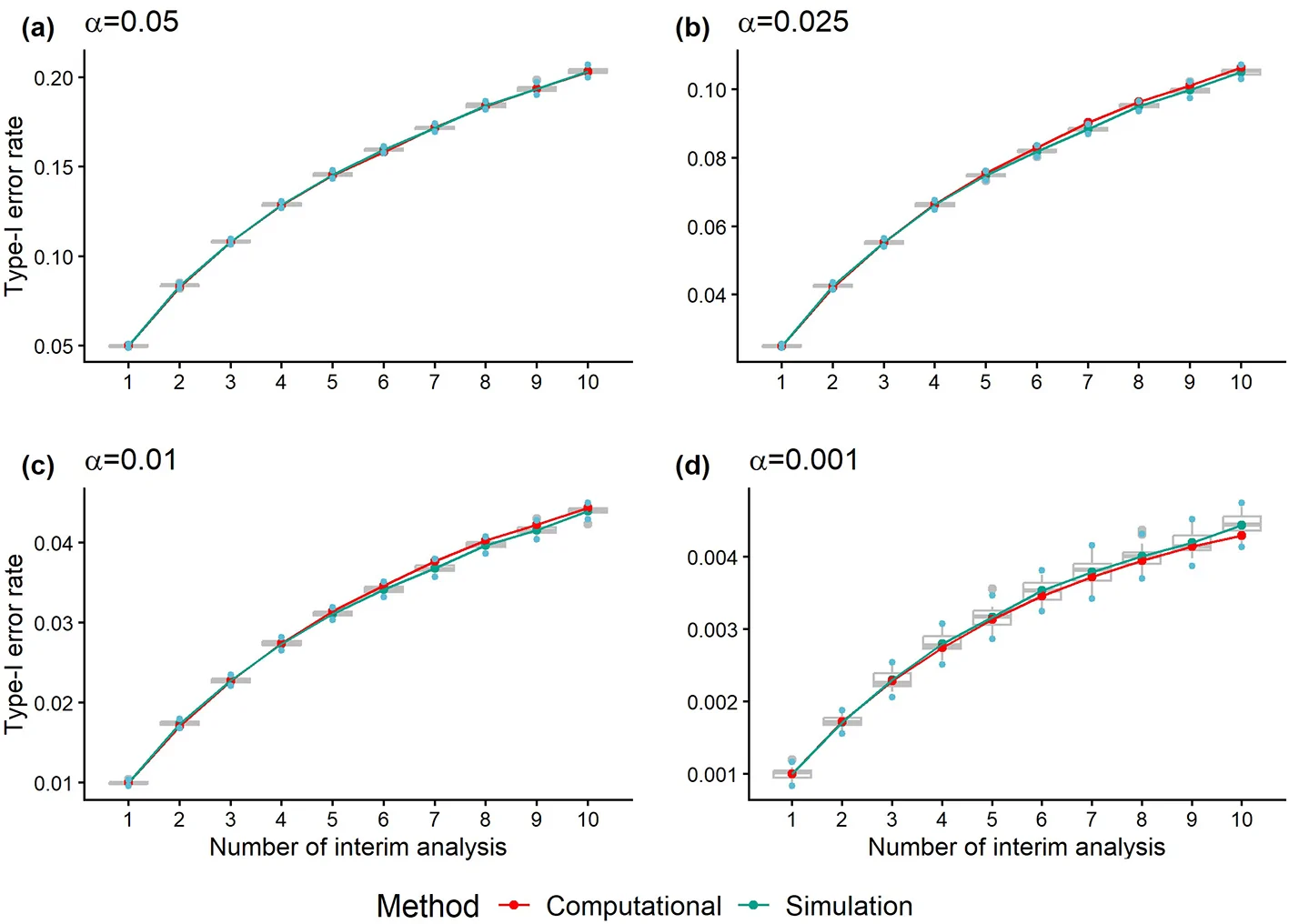
Computational and simulation models' performance for the *n* = 320 condition. The box plots and the green line indicate the simulated points and their mean values. The two blue dots represent the two standard deviations from the simulated mean, while the red lines indicate the computational points. **(a)** α = 0.05; **(b)** α = 0.025; **(c)** α = 0.01; **(d)** α = 0.001.

To account for simulation errors, the simulation study was conducted twenty times, and the resulting set of TIE rates was recorded. For each simulation design cell (*n*, *k*, α), the computational model was reliable in estimating the TIE rate when its value was within two standard deviations from the simulated mean values. However, when its values exceeded the two standard deviations, its performance did not agree with the values generated by the MC simulation model, which played the role of *gold standard* in our validation context. Figures 1–4 show the results of our analysis. The four graphs in each figure show the computational model's performance as a function of the different α values.

The results highlight the good performance of the computational model across a variety of sample sizes (40, 80, 160 and 320) and significance levels (.001, .01, .025, .05). Overall, the model has demonstrated the ability to predict TIE rates, remaining consistent with the simulation results in almost all tested conditions. In particular, all values estimated by the computational model fall within two standard deviations from the mean of the simulated points. Regardless of the sample size, the TIE rate increases with the number of interim analyses, a behavior that is expected and reflects the nature of the multiple testing process. This trend was clearly observed in all graphs, indicating that the model is capable of correctly capturing the relationship between the number of interim analyses and the TIE rate. One of the most relevant observations concerns the model's ability to maintain its accuracy even with smaller sample sizes. In cases with sample sizes of 40 and 80, where intrinsic variability is higher, the computational model's line (red) continued to significantly overlap with the simulation boxplots. This suggests that the model is robust and can be used in studies with limited sample sizes without losing reliability. Furthermore, the model performed well across a range of significance levels. For α =.025 and α =.05, the results are particularly convincing, with the model's line remaining predominantly within the simulation boxplots. Even for more stringent significance levels such as α =.001 and α =.01, the model maintained good alignment with the simulation data. Finally, for high values of *k* (number of analyses computed) and lower values of α a little departure of approximate values from the simulated mean was observed while staying within the two standard deviations. This result is likely due to an approximation bias provided by built-in *R* functions adopted to compute integrals, particularly when smaller α values entailed truncated distributions at more extreme values. Furthermore, for very small α values, the tail of the simulated probability density function contained only a few observations, thus contributing to a larger degree of variability. By contrast, when the α value was larger, we observed computational values closer to the simulated mean since the cut-off point was closer to the distribution mean and more observations were included in the distribution tail.

## 4 Discussion

In this article, we proposed a new computational model to estimate the effect of the DP practice in the growth of TIE rate. We validated this model by comparing its performance with the simulation approach which played the role of a *gold standard* model in the evaluation study. Both simulation and computational models assume a null hypothesis of no effect when applied in a one-sample t-test scenario. We also varied three factors (total sample size, number of analysis, and TIE probability) in a full factorial MC simulation design. Overall, the results show that the proposed computational model performs well. Across all conditions, its estimates consistently fell within two standard deviations of the simulated means, indicating that the model offers an efficient approximation of the TIE rate induced by DP practice. The observed findings are also consistent with previous literature showing that DP practices substantially inflate the probability of obtaining statistically significant results even in the absence of a true underlying effect (Stefan and Schönbrodt, 2023; Armitage et al., 1969). Across all tested conditions, the TIE rate increased beyond the nominal α level, demonstrating how even a relatively small number of interim analyses may considerably distort statistical inference. These findings further support concerns that DP practices may enable researchers to obtain false positive significant results with relative ease through repeated analytical decisions during data collection (Ioannidis, 2005, 2008; Button et al., 2013). More generally, the present findings highlight how even relatively limited forms of repeated interim testing may substantially distort statistical inference when appropriate correction procedures are not applied.

The obtained results have both theoretical and practical implications. Theoretically, our model provides a tool for analyzing TIE rate in the context of DP practice, allowing for a more in-depth examination of how increasing the number of interim analysis influences this error. Traditional simulation studies (e.g., Simmons et al., 2011; Roettger, 2019; Stefan and Schönbrodt, 2023) may, indeed, become increasingly challenging when multiple experimental factors must be explored (Browne et al., 2025). The proposed computational model overcomes this limitation bypassing the need for Monte-Carlo simulation entirely. Instead of approximating the TIE rate through repeated data simulation, the model directly applies the mathematical expression describing the DP procedure. This allows us to estimate TIE rate using functions that can be easily implemented in R, avoiding the computational burden and stochastic uncertainty inherent in simulation-based models. As a result, it becomes feasible to explore a greater number of factors influencing the increase in the TIE rate due to interim analysis practices. Practically, the proposed computational model empower researchers to avoid the pitfalls associated with traditional simulation approaches. It deletes Monte-Carlo simulation error, effectively deleting the issue of the limited number of simulations often found in the literature (Morris et al., 2019). Furthermore, the proposed model minimizes the possibility of human errors during the construction of simulations, which in turn reduces the time needed to design experimental studies. By increasing the number of factors that can be explored and minimizing setup errors, our method ensures a more reliable (in terms of reducing the error component) and efficient analysis.

Although a direct comparison with existing sequential correction procedures falls beyond the primary scope of the present work, the connection with the FWER literature is important to clarify the distinction between planned interim analyses and questionable DP practices. In planned sequential designs, procedures such as Bonferroni corrections, group-sequential methods, and alpha-spending approaches are specifically intended to control the overall TIE rate across repeated analyses. By contrast, the present work focuses on the uncorrected form of repeated interim testing typically associated with DP practices, in which data collection is repeatedly monitored and stopped once statistical significance is observed without applying any adjustment to the nominal significance threshold. In this context, the proposed computational framework should not be interpreted as an alternative to existing sequential correction procedures, but rather as a tool for quantifying the inflation of the TIE rate produced by uncorrected DP practices under different interim analysis and sample size conditions. However, because the proposed model explicitly models the relationship between the number of interim analyses, sample size, and TIE-rate inflation, future research could investigate whether it may be extended to derive sequential testing procedures, thereby providing a more direct comparison with existing FWER-control methods. Furthermore, the model presented in this study focuses on a single p-hacking practice while the broader landscape of p-hacking is extensive, encompassing many techniques that can affect results in complex and interconnected ways. As QRPs can compromise reliability of methodological and assessment procedures, they are especially relevant in fields like Educational Sciences, Medicine, Psychology, Neuroscience, and clinical Neuropsychology, in which high-stake decisions might be taken based on the results of assessment (see, e.g., Ioannidis, 2005; Button et al., 2013; Gelman and Geurts, 2017. In these contexts, analytical flexibility, repeated testing, and selective reporting practices may contribute to the inflation of false positive findings and exaggerated effect sizes, ultimately reducing the replicability and credibility of empirical evidence (Ioannidis, 2005, 2008). For this reason, future work should expand the current model to incorporate multiple QRPs simultaneously and to account for their potential interactions. To support such developments, this article outlines two fundamental steps required to build computational models that quantify how p-hacking practices inflate the TIE rate. The first step is to identify the mathematical expression that characterizes the outcome produced by each specific QRP. For instance, under the DP practice, the final result can be expressed as the weighted mean of the collected datasets (Equation 5). Once this expression is established, the second step involves formulating it in terms of probability density functions. In the case of DP, the probability density function of the resulting statistic can be obtained through the convolution of the probability density functions of the means of the individual datasets involved (Equations 15 and 16). This framework allows one to mathematically model how DP reshape the distribution of results, thereby shifting outcomes toward false positives. Moreover, because the practical impact of QRPs is likely to vary substantially across different research contexts, future extensions of the proposed framework could incorporate prior probabilities and contextual information related to different research settings. Such developments may allow future models not only to simultaneously account for multiple QRPs, but also to estimate how their combined effects vary across different research contexts.

Since extending the model to other QRPs or to scenarios where multiple practices are applied simultaneously is likely to be challenging, the main limit of the present work lies in its restricted generalizability. While the model provides useful insight into DP practice within the simple framework of a one-sample t-test, its application beyond this context is less straightforward. Formalization of DP is feasible because this practice follows a recurrent and sequential structure. In DP, the researcher performs a fixed cycle of actions: collect data, test for significance, then either stop or continue. This ordered repetition makes it possible to express the resulting probability density function through an analytic derivation that mirrors the step-by-step development of the DP procedure, making explicit the connection between interim results and the final outcome (see Equation 3). However, many p-hacking practices do not follow such a clear or repetitive structure, making the derivation of the corresponding probability distributions non-trivial, and this difficulty increases considerably when multiple practices are combined. Furthermore, extending the approach to more complex statistical frameworks would lead to substantially different distributional properties, thereby making the derivation of probability density functions under QRPs even more challenging.

## Data Availability

The datasets generated for this study can be found at: https://github.com/FrancescaFreuli/New-Frontiers-P-hacking.
